# Classification of tungsten-containing oxidoreductases provides insights into their biochemical and physiological diversity

**DOI:** 10.3389/fmicb.2026.1849799

**Published:** 2026-06-19

**Authors:** Saisuki Putumbaka, Michael P. Thorgersen, Gerrit J. Schut, Farris L. Poole, Claire E. Barrow, Jennifer B. Glass, Michael W. W. Adams

**Affiliations:** 1 Department of Biochemistry & Molecular Biology, University of Georgia, Athens, GA, United States; 2 School of Earth and Atmospheric Sciences, Georgia Institute of Technology, Atlanta, GA, United States

**Keywords:** aldehyde oxidation, human gut microbiome, iron sulfur cluster, oxidoreductase, tungsten

## Abstract

Tungsten-containing oxidoreductases (WORs) are a diverse family of enzymes with over 4,000 known members that can be subdivided into 92 clades based on the phylogeny of the large pyranopterin cofactor-containing large subunit (WorL). Despite being widespread in Bacteria and Archaea, particularly in members of the human microbiome, only five of the 92 WOR clades contain a WOR with a defined physiological role in cellular metabolism, primarily—but not exclusively—involved in oxidation of various aldehydes. However, this phylogenetic-based organizational system lacks a perspective on the diversity and complexity of WOR enzymes. Herein, we propose a non-phylogenetic classification system for WORs based on predicted subunit composition and electron carrier specificity that provides insight into potential physiological roles. WORs can be divided into five classes that range in complexity and predicted function. The simpler cytoplasmic Class I-III WORs are involved in aldehyde detoxification, a modified glycolysis pathway and cold adaptation. More complex multimeric WORs are proposed to use multiple electron carriers in bifurcating reactions (Class IV) or interact with various respiratory systems via associations with the cell membrane (Class V). We characterized two new WORs, one Class I and one Class V, from the human gut bacterium *Cetobacterium somerae*, and showed that the former enzyme had aldehyde oxidation activity but the latter did not. By combining phylogenetic information with the new WOR classification system, we can predict structural and functional characteristics of as-yet uncharaterized WORs and identify unique and novel enzymes for future studies.

## Introduction

The human gastrointestinal microbiome impacts human health in many ways, from nutrient absorption to metabolism to immune response. Gut microbiome composition is correlated to Alzheimer's disease, liver disease, inflammatory bowel disease, Parkinson's, and many others human health related conditions (Hall et al., 2017; NIH_Human_Microbiome_Portfolio_Analysis_Team, 2019; Rosas-Plaza et al., 2022; Bradley and Haran, 2024; Mishra et al., 2024; Wilson et al., 2024). To better understand human gut microbes and the mechanisms by which they can affect human health, we must better understand their biochemical and metabolic processes.

Recent studies have revealed the potential importance of the group six element, tungsten (W), in the gut microbiome. W and the analogous group six element molybdenum (Mo) are found in their oxyanionic forms in biological systems as tungstate (${\text{WO}}_{4}^{2-}$) and molybdate (${\text{MoO}}_{4}^{2-}$) and are incorporated into pyranopterin cofactors that are utilized in the active sites of a variety of enzymes. Enzymes that contain the molybdopyranopterin cofactor (Moco) are ubiquitous in prokaryotes and eukaryotes, including humans (Hille et al., 2014). Over fifty Moco-containing enzymes have been structurally and functionally characterized. They are classified into three major phylogenetically distinct families termed dimethyl sulfoxide reductase (DMSOR), xanthine oxidase (XO), and sulfite oxidase (SO).

Tungsten-containing oxidoreductases (WORs) are a distinct family of enzymes that are widely distributed in bacteria and archaea (over 4,000 UniProt sequences). To date, characterized members of the WOR-family carry out reactions classified as EC 1.2.7.5 and 1.2.7.6 (Bairoch, 2000). Interestingly, so far no W-containing proteins have been identified in eukaryotic organisms (Szaleniec and Heider, 2025). WORs can be phylogenetically subdivided into 92 clades based on the sequence of the large subunit, WorL, yet only five of those clades contain a functionally characterized member (Schut et al., 2021). Characterized WORs are diverse in subunit composition and display a range of physiological functions and electron carrier specificities.

To better understand the roles of WOR enzymes, we developed a non-phylogenetic classification system based on predicted subunit compositions and electron carrier specificities. We discuss this classification system in terms of previously characterized WOR enzymes and demonstrate its utility by the characterization of two WOR enzymes from the human gut bacterium *Cetobacterium somerae*. By combining WOR phylogenetic information and the new WOR classification system, we can predict structural and functional characteristics of uncharaterized WORs and target enzymes likely to have novel biochemical reactions or physiological roles for future studies.

## Results and discussion

### Classification of members of the WOR family

**Figure 1:**
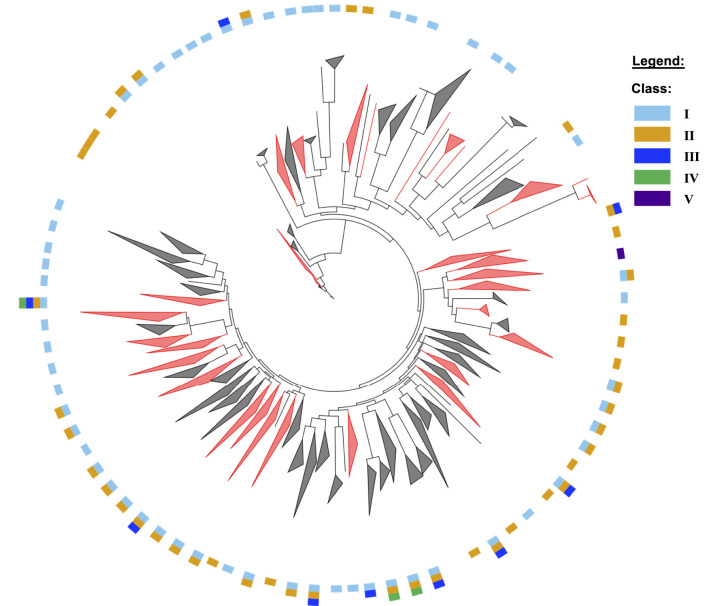
Phylogenetic tree of WOR family with >4,000 proteins and class designation of each. This phylogenetic tree was previously generated as part of the work done by Schut et al. (2021) and the family has been truncated and subdivided into 92 clades. Classes found in each clade are designated in the outer ring with Class I WORs shown in light blue, Class II WORs shown in yellow, Class III WORs shown in dark blue, Class IV WORs in green, and Class V WORs shown in purple. Class V WORs are only found in clade 41 while Class I and Class II are the most widespread. Clades that have representatives that are found in the human gut microbiome are designated in red. Additionally, several clades (24, 32, 37, 39, and 59) do not have a Class designation as they are now obsolete InterPro sequences.

We analyzed the gene neighborhoods of 4,064 WorL sequences from UniProt indentified by their containing two domains: the N-terminal domain (IPR013983) and C-terminal (IPR001203) domain (Figure 1, Supplementary Dataset 1). Using the EFI Gene Neighborhood Tool (EFI-GNT) (Zallot et al., 2019; Oberg et al., 2023) and what is known about the characterized members, we divided WOR enzymes into Classes I-V, as described below. Since the initial study of this type (Schut et al., 2021) of the WOR family some sequences are obsolete or are no longer recognized as valid WorL subunits by the current InterPro classification standards (Supplementary Dataset 2) including clades 24, 32, 37, 39, and 59. Nevertheless, the original 92-clade phylogenetic tree (Schut et al., 2021) is a valid representation of the diversity found within the WOR family and was used for the classification of WORs. Despite functional and quaternary structure diversity among WOR family members, their secondary and tertiary structures for the L subunits are highly conserved with very little variation in the amino acids that coordinate the tungstopyranopterin, especially in most aldehyde-oxidizing members (Thr243, Glu313, Tyr427, and His448, using PfAOR numbering), and [4Fe-4S] cofactors. The predicted folds of the L subunit closely align with experimentally-determined structures (Szaleniec and Heider, 2025).

### Class I WORs

#### Previously characterized Class I WORs

**Figure 2:**
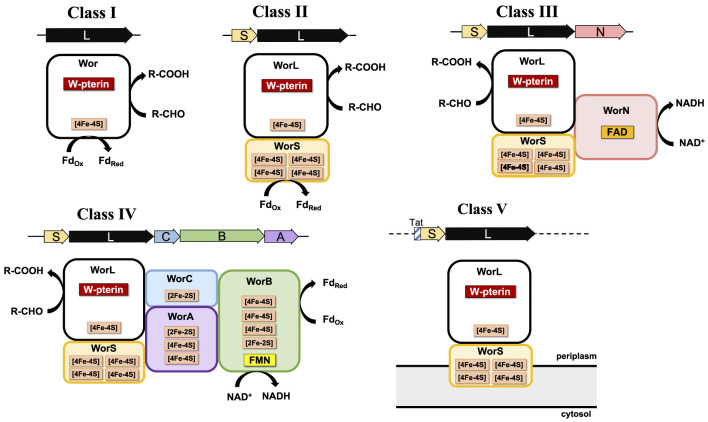
Classification of WORs. The subunit composition of each class of WOR is shown where the large, tungstopterin containing subunit (L, black) is common to all classes, and the small subunit (S, yellow) is common to four of the classes. Fd_ox_ and Fd_red_ represent the oxidized and reduced forms of ferredoxin, respectively. Class V WORs have various subunit compositions, but all have the S and L subunits of the Class II enzymes but the S has a Tat signal and these are membrane associated enzymes.

Class I WORs consist solely of a large subunit (WorL) containing the tungstopyranopterin (Tuco) catalytic site and one [4Fe-4S] cluster (Figure 2). Some Class I WORs encode an additional “ThiS-like” protein nearby that we term WorX. Although its exact function is unknown, we predict that WorX is involved in pterin-biosynthesis. Four Class I WORs [PfAOR, PfGAPOR, BmWOR, and CsWOR87 (this study)], have been biochemically characterized, each of which has different substrate specificities, but all of which transfer electrons to ferredoxin (Fd) and appear to be involved in aldehyde oxidation. The majority of WORs (55/92 clades) belong to Class I (Tables 1, 2).

**Table 1:** Classification of WOR family.

Class	Clade
I	6, 7, 8, 9, 10, 11, 12, 13, 14, 15, 16, 17, 18, 21, 22, **23**, 25, 26, 27, 28, 29, 30, 34, 36, 38, 43, 44, 47, 49, 56, 57, 65, 66, 70, 71, 72, 73, 74, 75, 76, 77, 78, 79, 80, 82, **83**, 84, 85, 86, **87**, 88, 89, 90, 91, 92
II	1, 2, 3, 4, 5, 6, 7, 19, **20**, 31, 33, 35, **40**, 42, 45, 46, 48, 49, 50, 51, 52, 53, 54, 55, 57, 58, 60, 61, 64, 67, 68, 69, 70, 71, 74, 75, 76, 77, 81, 87
III	13, 67, **87**
IV	**62**, 63, **87**
V	41

The clades represented within each class are listed. Clades in bold have characterized representatives. Clades in red have members found in the human gut microbiome. Clades 6, 7, 13, 57, and 87 contain members from more than one class.

**Table 2:** Distribution of classes in the WOR family phylogenetic tree (Figure 1).

Class	Subunits	Electron carriers	Characterized members (clade)	Physiological role	Class features	Clades (of 92)	Total	%
I	WorL	Fd	PfAOR (87) BmWOR (83) PfGAPOR (23) CsWOR87 (87)	Aldehyde detoxification Aldehyde detoxification Glycolysis Glycolysis		55	2,341	65.4
II	WorL WorS	Fd	PfASOR (40) CbGOR (20)	Aliphatic sulfonate oxidation Glycolysis		40	574	16.0
III	WorL WorS WorN	NAD^+^	AaAOR (87)	Aldehyde oxidation for growth	Nanowires	9	179	5.0
IV^*^	WorL WorS WorA WorB WorC	Fd NAD(P)^+^	ElWOR1 (87) AmWOR1 (87) GmBCR (62)	Aldehyde detoxification Aldehyde detoxification Benzoyl-CoA reduction	Electron bifurcation Nanowires^**^	1	24	2.9
V^*^	WorL WorS	Electron transport chain	EcYdh (41) CsWOR41 (41)	Unknown Unknown	Membrane associated	1	380	10.6

The WOR enzyme classes as defined by subunit composition and electron carrier specificity. Characterized members are listed with phylogenetic tree clade in parentheses. Known physiological functions and common features for class members are listed. The number of clades containing members of each class out of 92 total clades is listed along with the total number of WORs for each class and the percentage of the total number of WORs in each class.

^*^Some members of these classes contain a variety of additional subunits.

^**^GmBCR does not contain nanowires.

Aldehyde oxidoreductase (PfAOR, clade 87) from the hyperthermophilic anaerobic heterotrophic archaeon *Pyrococcus furiosus* (Pf) was the first WOR to be characterized (Mukund and Adams, 1991). PfAOR oxidizes a range of aliphatic and aromatic aldehydes, including acetaldehyde and phenylacetaldehyde (Mukund and Adams, 1991). Its function *in vivo* is likely detoxification of aldehydes generated during peptide fermentation, as it is most active with aldehydes derived from the decarboxylation of 2-ketoacids generated by the transamination of amino acids. The apparent K_m_ of PfAOR for PfFd is 10 μM, consistent with Fd being the native electron carrier. The structure of PfAOR, the first of any pyranopterin-containing enzyme (with either W or Mo), showed that it is a homodimer that is bridged by a single Fe atom (Chan et al., 1995). Of the characterized Class I clade 87 WORs in Thermococcales species, this homodimer structure is unique to PfAOR, which has unique residues (Glu332 and His383) that bind the Fe^2+^ atom and bridges the homodimer together (Szaleniec and Heider, 2025).

Glyceraldehyde-3-phosphate ferredoxin oxidoreductase (PfGAPOR, clade 23), also from *P. furiosus*, was the second Class I WOR to be characterized (Mukund and Adams, 1995). PfGAPOR is specific for glyceraldehyde 3-phosphate with high affinity (*K*_*m*_ = 28 μM) and has high affinity for PfFd (*K*_*m*_ < 6 μM) (Mukund and Adams, 1991, 1995). PfGAPOR replaces glyceraldehyde 3-phosphate dehydrogenase (GAPDH) in a modified glycolytic pathway in *P. furiosus* (Mukund and Adams, 1995) in which the product of glyceraldehyde 3-phosphate oxidation by PfGAPOR is 3-phosphoglycerate rather than the 1,3-bisphosphoglycerate produced by GAPDH.

We recently characterized a third member of Class I WORs from the obligately aerobic bacterium *Brevibacillus massiliensis* (Bm), isolated from human feces (Hugon et al., 2013; Thorgersen et al., 2022). The BmWOR (clade 83) was the first WOR purified from an obligate aerobe. BmWOR is monomeric and has highest specific activities on aromatic aldehydes, such as benzaldehyde, cinnamaldehyde and tolualdehyde (K_m_ values < 6 μM, k_cat_/K_m_ > 1,400 mM^−1^ s^−1^), suggesting that it detoxifies food-derived aldehydes that the bacterium encounters in the gut environment (Thorgersen et al., 2022). In contrast to all other members of the WOR family characterized so far, it is catalyticaly active in air using BmFd (*K*_*m*_ < 5 μM) as the electron acceptor (Thorgersen et al., 2022; Szaleniec and Heider, 2025). All WORs from Clade 83 are Class I, with some found in aerobic halophilic archaea and others in thermophilic bacteria. Several clade 83 Class I WORs are associated with human gastrointestinal microbes (Thorgersen et al., 2022).

#### Characterization of a new Class I WOR from the gut bacterium *Cetobacterium somerae*

*C. somerae* is a rod-shaped, gram-negative, strictly anaerobic, non-spore forming bacterium isolated from human feces (Finegold et al., 2003). We recently sequenced, assembled, and annotated its 3.2 Mb genome, which consists of one chromosome and seven plasmids (Putumbaka et al., 2025a). Genome analysis of *C. somerae* revealed two members of the WOR family representing Clades 41 and 87 (ACIE8Z_02170 and ACIE8Z_02065), as well as the W-transport system TupABC (ACIE8Z_02150, ACIE8Z_02155, and ACIE8Z_02160) all of which are found on the largest plasmid (plasmid 01, 687,705 bp) (Putumbaka et al., 2025a). These WORs will be referred to as CsWOR41 and CsWOR87, respectively (Supplementary Figure S1). CsWOR87 is a Class I WOR, consisting of a single large subunit adjacent to *worX* and *thiF*, which are thought to be involved in pterin cofactor biosynthesis rather than subunits of the enzyme (see below for details on CsWOR41, a Class V WOR). The *C. somerae* genome also encodes the molybdate transporter, ModAB (ACIE8Z_14485 and ACIE8Z_14490 encoding a fused ModBC) and six Mo-containing enzymes, four from the DMSOR family (ACIE8Z_07505, ACIE8Z_11150, ACIE8Z_01885, and ACIE8Z_03800), and two from the XO family (ACIE8Z_10510 and ACIE8Z_01375).

**Figure 3:**
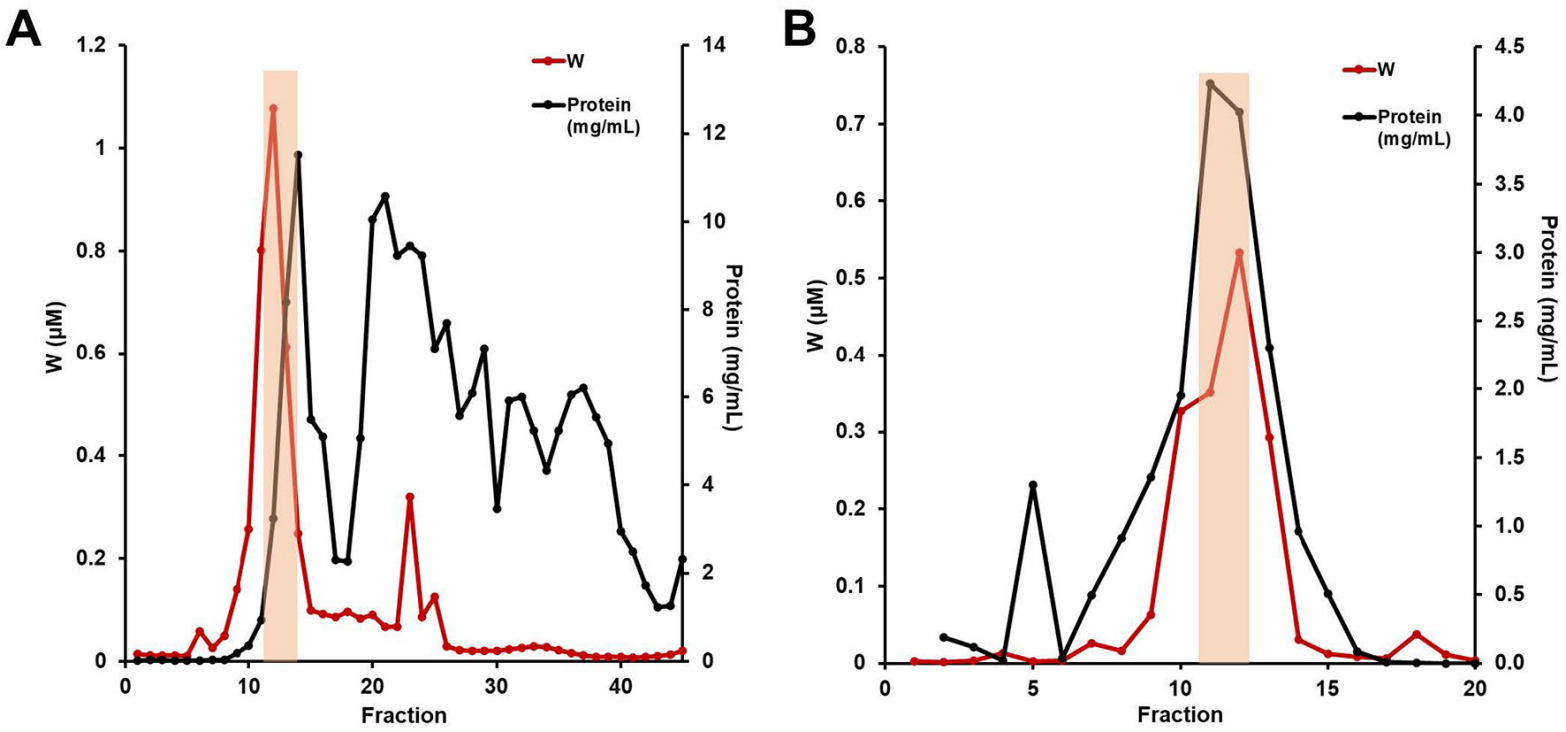
*C. somerae* tungsten incorporation and CsWOR87 activity. **(A)** *C. somerae* cytoplasmic extract was separated using an anion exchange chromatography column (QHP). W (red), and protein (black) concentrations within each fraction are shown. The orange box highlights the fractions (12–14) that had furfural oxidation activity, which corresponds with the major W-peak. **(B)** Fractions (12–14) of the anion exchange column were subsequently separated on a size exclusion column (Superdex 200). W (red), protein (black) concentrations within each fraction are shown. The orange box highlights the active fractions (11–12) that had activity with furfural.

To provide further insight into the WORs in *C. somerae*, we attempted to purify them from native biomass. *C. somerae* cells were grown with 100 nM ${\text{WO}}_{4}^{2-}$ and 100 nM ${\text{MoO}}_{4}^{2-}$ added. The cytoplasmic extract from 15 g of cells was fractionated by anion exchange chromatography (QHP) under anoxic conditions and the resulting fractions were analyzed for their metal contents using inductively coupled plasma mass spectrometery (ICP-MS) (Supplementary Figure S2). *C. somerae* cytoplasmic proteins contained about 7-fold higher Mo than W (~176 vs. 25 μmoles g^−1^; Supplementary Figure S2). Furfuraldehyde oxidation activity was associated with the major peak of W content in fractions (12–14) from the QHP column (Figure 3), with the highest dye-linked furfural oxidation activity in fraction 12 (0.31 U/mg, Supplementary Table S1). The active fractions were pooled and further purified using size exclusion chromatography and CsWOR87 was identified (in fractions 11 and 12) by liquid chromatography-tandem mass spectrometry (LC-MS/MS) analysis (Figure 3B). We conclude that the furfuraldehyde oxidation activity is associated with CsWOR87 as no other enzyme with such an activity (e.g., aldehyde dehydrogenase, CsWOR41), was detected in the same fractions. CsWOR87 has broad substrate specificity and is active with various aliphatic and aromatic aldehydes (Supplementary Table S1), some of which are food-derived and/or have been identified in the gut metabolome (Schut et al., 2021).

### Class II WORs

Class II WORs contain WorL and a second small subunit (WorS) (IPR017896) with four [4Fe-4S] clusters, and use Fd as an electron carrier (Figure 2). Class II WORs are the second most common (16% of the total) and are found in nearly half of the WOR clades (40 of 92; Tables 1, 2). Two Class II WORs have been characterized: an aliphatic sulfonate-oxidizing enzyme from *P. furiosus* (PfASOR; clade 40) and a glyceraldehyde-3-phosphate-oxidizing from the thermophilic cellulolytic bacterium *Caldicellulosiruptor bescii* (CbGOR; clade 20). PfASOR was originally characterized as a WorL homodimer without WorS and shown to oxidize a wide range of aliphatic and aromatic aldehydes *in vitro* [highest activity with hexanal; V_m_ = 15.6 μmoles min^−1^ mg^−1^, K_m_ = 180 μM, k_cat_/K_m_ = 188 mM^−1^ s^−1^ (Bevers et al., 2005)]. Cyclic voltammetry indicated that Fd was the native electron carrier. More recent purification resulted in a heterodimeric PfASOR containing both WorL and WorS subunits. From the X-ray structure the electron density in the active site was modeled as an aliphatic sulfonate (Mathew et al., 2022). Biochemical assays confirmed that PfASOR was active with the aliphatic sulfonate taurine [V_m_ of 14.9 μmoles min^−1^ mg^−1^, an apparent K_m_ 112 μM, and k_cat_/K_m_ = 288 mM^−1^ s^−1^ (Mathew et al., 2022)]. PfASOR expression is upregulated as part of the cold shock response. It is now proposed that PfASOR is involved in the oxidation of various aliphatic sulfonates and phosphonates during adaptation to lower growth temperatures (Mathew et al., 2022). Like PfGAPOR (Scott et al., 2019), CbGOR oxidizes glyceraldehyde 3-phosphate in place of GAPDH in glycolysis and uses Fd as an electron acceptor (K_m_ of 38.9 μM). Unlike monomeric PfGAPOR, CbGOR is a heterodimer [WorLS; (Scott et al., 2019)].

### Class III WORs

Class III WORs contain a third subunit (here, termed WorN: IPR004982 or IPR023753) in addition to WorLS. WorN contains an FAD-binding site for electron transfer to and from NAD^+^. Class III WORs are found in only 9 of the 92 clades and make up 5% of the WOR family (Tables 1, 2). To date, only one Class III WOR has been characterized: AaAOR (clade 87) from the mesophilic bacterium *Aromatoleum aromaticum* EbN1 (Aa) (Arndt et al., 2019). All three subunits (WorLSN) were observed in natively purified AaAOR, which oxidizes aromatic and aliphatic aldehydes using NAD^+^ as an electron acceptor (apparent K_m_ of 12.5 μM). Although *A. aromaticum* is a strict anaerobe, AaAOR was somewhat resistant to O_2_ exposure (1 hr half-life after air exposure), in contrast to BmWOR, which retains 50% of activity after 48 h in air (Arndt et al., 2019; Thorgersen et al., 2022). AaAOR production is induced when *A. aromaticum* is grown on phenylalanine and it is proposed to degrade toxic aromatic aldehyde intermediates, such as phenylacetaldehyde, generated during growth on aromatic amino acids (Arndt et al., 2019). More recently, a structure was obtained, predominantly with (LS)_3_N stoichiometry, in which the LS subunits were shown to form a “nanowire” type structure enabling electron transfer from the aromatic aldehyde to NAD^+^ (Winiarska et al., 2023).

### Class IV WORs

Class IV enzymes have the same small and large (WorLS) subunits as Class II and III WORs, but contain an electron bifurcation module of three additional subunits (WorABC) that allow for the enzyme to simultaneously reduce both ferredoxin and NAD(P)^+^ (Feng et al., 2025). Class IV WORs make up less than 1% of the WOR family (Tables 1, 2). ElWOR1 from the mesophilic gut bacterium *Eubacterium limosum* oxidizes a wide range of aromatic and aliphatic aldehydes with a proposed physiological role of acetaldehyde detoxification during lactate metabolism (Putumbaka et al., 2025b).

The electron-bifurcating AmWOR1 from the thermophilic bacterium *Acetomicrobium mobile* (Am) oxidizes a wide range of aliphatic and aromatic aldehydes (Schut et al., 2021). In the cryo-EM structure, the three WorSL subunits form [4Fe-4S] cluster “nanowires” that funnel electrons from aldehyde oxidation into a single WorABC electron-bifurcating “plug” that simultaneously reduces Fd and NAD^+^ (Schut et al., 2021; Feng et al., 2025). This mechanism may allow for metabolic buffering, where the organism is able to maintain a consistent flux of high-energy, low-potential electrons even when substrate availability fluctuates. Additionally, the bifurcation mechanism may allow for the detoxification of aldehydes and cycling of electrons when aldehyde concentration is extremely low even in the presence of a high concenteration of the corresponding acid. Specifically, since the reduction potential of the aldehyde/acid redox couple is approximately the same as that of Fd (E ~ −500 mV), aldehyde oxidation coupled to Fd reduction at low concentration and a high aldehyde:acid ratio is only energetically favorable if it is also coupled to the exergonic reduction of NAD^+^ (E ~ −280 mV).

The benzoyl-CoA reductase (GmBCR; clade 62) from *Geobacter metallireducens* consists of eight distinct subunits (BamBCDEFGHI) with a (BC)_2_DEFGHI stoichiometry from the cryo-EM structure (Weinert et al., 2015; Huwiler et al., 2019). Interestingly, the GmBCR structure does not show nanowire features like other Class IV members, but BamB is equivalent to WorL and BamC to WorS but with three rather than four [4Fe-4S] clusters. The BamIHG subunits are homologous to the WorABC subunits. BamE and D are unique subunits that show homology to the non-hydrogenase subunits HdrA, B and C in the bifurcating MvhAGD-HdrABC hydrogenase complexes found in methanogens (Seelmann et al., 2020; Schut et al., 2022). GmBCR is the only known WOR with non-aldehyde linked activity as it reduces an aromatic ring driving the reduction of benzoyl-CoA to dienoyl-CoA (Huwiler et al., 2019). These Class IV WORs occur in clades 62 and 63, mainly in organisms that degrade aromatic compounds.

**Figure 4:**
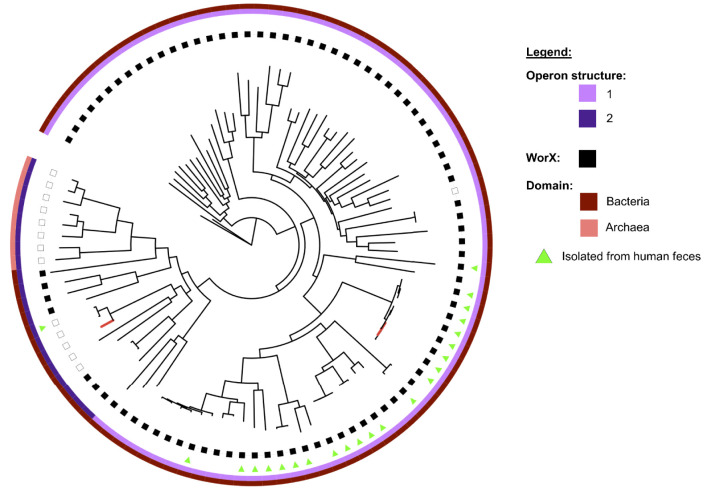
Phylogeny of Class IV WORs. Bifurcating WORs (102 sequences) from Clade 87 were identified through EFI enzyme similarity and gene neighborhood tools (Zallot et al., 2019). Of these, there are two different operon structures: (1) SLCBA (light purple) and (2) CBASL (dark purple) as depicted in Supplementary Figure S3. Whether the protein is from a Bacteria (dark red) or Archaea (light red) are annotated. Additionally, gene clusters that have a *worX* gene, which is not a subunit but rather thought to be involved in pterin cofactor processing, is also designated with a black square. The green triangles indicates a gut-related organism and red nodes indicate *A. mobile* and *E. limosum*.

We identified 102 unique Class IV WORs in EFI-GNT gene neighborhoods (Zallot et al., 2019; Oberg et al., 2023), 23 of which are present in bacteria in the human gut (Figure 4). Intriguingly, these included methanogenic archaea from the genus *Methanofollis*, which use alcohols as an electron donor and their bifurcating WOR is likely involved in alcohol metabolism (Imachi et al., 2009). We identified two operon structures. Type 1 operon has the gene order WorSLCBA while the type 2 operon has a WorCBASL orientation. The WOR of *A. mobile* is part of the type 2 operon while that of *E. limosum* has a type 1 structure (Schut et al., 2021). WorX is variably present within the operons, sometimes at the beginning, separating the SL and ABC, or at the end. Interestingly and for unknown reasons, WorX appears to be missing in the archaeal WORs cluster suggesting that these organisms may have a different pyranopterin maturation pathway.

### Class V WORs

#### Bioinformatic analysis of Class V WORs

**Figure 5:**
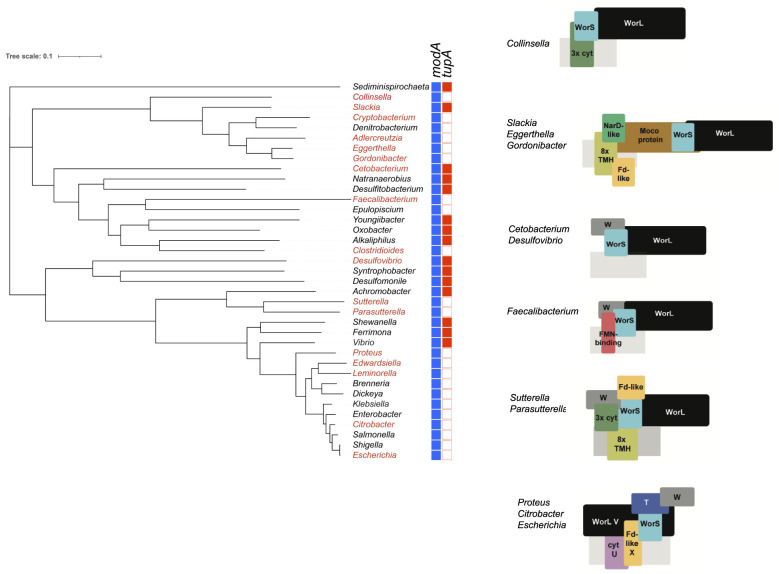
Distribution of Mo (*modA)* and W (*tupA)* transport genes in WOR41 (Class V) containing genera and the hypothetical subunit composition of Class V WORs from genera found in the human gut. A phylogenetic tree of genera containing a WOR41 (Class V) was constructed using multiple sequence alignments of COGs for 49 core universal genes (Price et al., 2010). For each genus, a single representative genome was selected for phylogenetic analysis (Supplementary Dataset 3). The presence or absence of *modA* and *tupA* for each genus was determined using BLASTp of known ModA and TupA protein sequences against taxonomic identifiers corresponding to the indicated genera (States and Gish, 1994). Genera with species that are commensal and found in the UHGG are in red.

Class V WORs are heteromultimeric enzymes associated with the cell membrane, suggesting that they serve respiratory roles. Over 10% of the WOR family members are Class V and they are found only in Clade 41 (Figure 5). All Class V WORs contain WorL, WorS and at least one other subunit, with a wide diversity of predicted subunit compositions (Figure 5). The periplasmic orientation of Class V WORs is indicated by a predicted twin arginine translocation (Tat) signal and/or a transmembrane domain in WorS (IPR006311). Class V WORs are present in facultatively anaerobic gammaproteobacteria (e.g., *Escherichia, Citrobacter, Proteus, Shewanella, Parasutterella, Sutterella*), obligately anaerobic gut-associated Coriobacteria (*Eggerthella, Gordonibacter, Slackia, Collinsella*), gut-associated and halophilic Clostridia (*Faecalibacterium, Oxobacter, Alkaliphilus, Natranaerobius*), Fusobacteriales (*C. somerae*), and sulfate-reducing Desulfobacterota (*Desulfovibrio, Syntrophobacter*; Figure 5).

The gene content of Class V WORs varies considerably (Supplementary Figure S4). Facultatively anaerobic Gammaproteobacteria encode respiratory-like enzymes containing subunits with multiple transmembrane domains as well as cytochrome-containing subunits. Coriobacteria possess a subunit with transmembrane domains as well as an unknown pyranopterin-containing subunit. Human gut dwelling Clostridia contain an unknown membrane-bound flavin-binding subunit. Respiratory-linked Class V WORs therefore give a completely new perspective on the range of physiological roles encompassed by WORs.

In *Escherichia coli*, the WorL and S subunits of its Class V WOR (EcYdh) are annotated as YdhV and YdhY and are part of the *ydh* operon that includes four other genes encoding YdhWXUT. However, *E. coli* is not known to utilize W and its genome encodes 19 Mo-containing enzymes (Iobbi-Nivol and Leimkuhler, 2013). Moreover, *E. coli* does not contain the high affinity tungstate transport system TupABC (Figure 5). This is in contrast to virtually all microbes that contain Class I-IV WORs and are assumed to incorporate W into their WORs. On the other hand, the molydate transporter ModABC in *E. coli* also transports tungstate, so it is possible that the Class V WOR in *E. coli* and in other species containing this class of enzyme but lacking TupABC do, indeed, insert W in their WORs. To provide insight into whether the Class V enzymes might utilize Mo rather than W, we analyzed all of the microbes that contain such enzymes for the presence of TupABC and ModABC transporters. As shown in Figure 5, the genomes of only 15 of the 37 genera containing Class V WORs encode TupABC, but all encode ModABC. Hence, it would seem likely that Class V WORs are unique in that some, and possibly the majority, are molybdoenzymes.

To date, no Class V WOR has been purified from a native host microbe and no biochemical activity or physiological role has been assigned to such an enzyme. The only relevant information comes from *E. coli*, where the gene (*ydhV*) encoding the WorL subunit is part of an operon encoding five additional subunits (Figure 5). In a previous study, the *E. coli ydhV* gene (but not the complete operon) was homologously over-expressed and purified using an affinity tag (Reschke et al., 2019). When the recombinant *E. coli* strain was grown in the presence of 10 μM ${\text{MoO}}_{4}^{2-}$ and 10 μM ${\text{WO}}_{4}^{2-}$, 40% of the purified L subunit contained Mo and W was not detected. In fact, significant amounts of W-loaded YdhV were only observed under growth conditions where the amount of ${\text{WO}}_{4}^{2-}$ added to the growth medium was over 10-fold higher than the amount of ${\text{MoO}}_{4}^{2-}$ added (Reschke et al., 2019). *E. coli* ModABC is known to have comparable affinities for tungstate and molybdate (Ge et al., 2020) and from this study (Reschke et al., 2019) it appears that its Class V WOR has a preference for Mo, but clearly W can also be incorporated. More definitive studies of *E. coli* using the holoenzyme are needed. To investigate whether other Class V WORs utilize W or Mo, we worked to experimentally characterize CsWOR41 from *C. somerae*.

#### Characterization of Class V WOR CsWOR41

The *C. somerae* genome contains a Class V WOR in a putative three-gene operon encoding CsWOR41LSW (Supplementary Figure S1). CsWOR41S has a Tat signal, indicating a periplasmic location. Genes encoding TatAE and TatC are found downstream of the CsWOR41 operon (Supplementary Figure S1) and are involved in transporting folded proteins with associated cofactors across the cytosolic membrane and into the periplasm (Hamsanathan and Musser, 2018). Upstream of the CsWOR41 operon are three genes encoding the tungstate specific transporter TupABC (ACIE8Z_02150, ACIE8Z_02155, ACIE8Z_02160). Downstream of the CsWOR41 operon there are genes encoding FdhD, a predicted sulfur transferase that might be required to produce active CsWOR41, and MoeA, involved in metal insertion into the pyranopterin cofactor (Thome et al., 2012). Notably, *C. somerae* contains two copies of MoeA, indicative of organisms that use both Tuco and Moco (Maia et al., 2016). The presence of TupABC directly upstream of the operon suggests that CsWOR41 is a tungstoenzyme.

**Figure 6:**
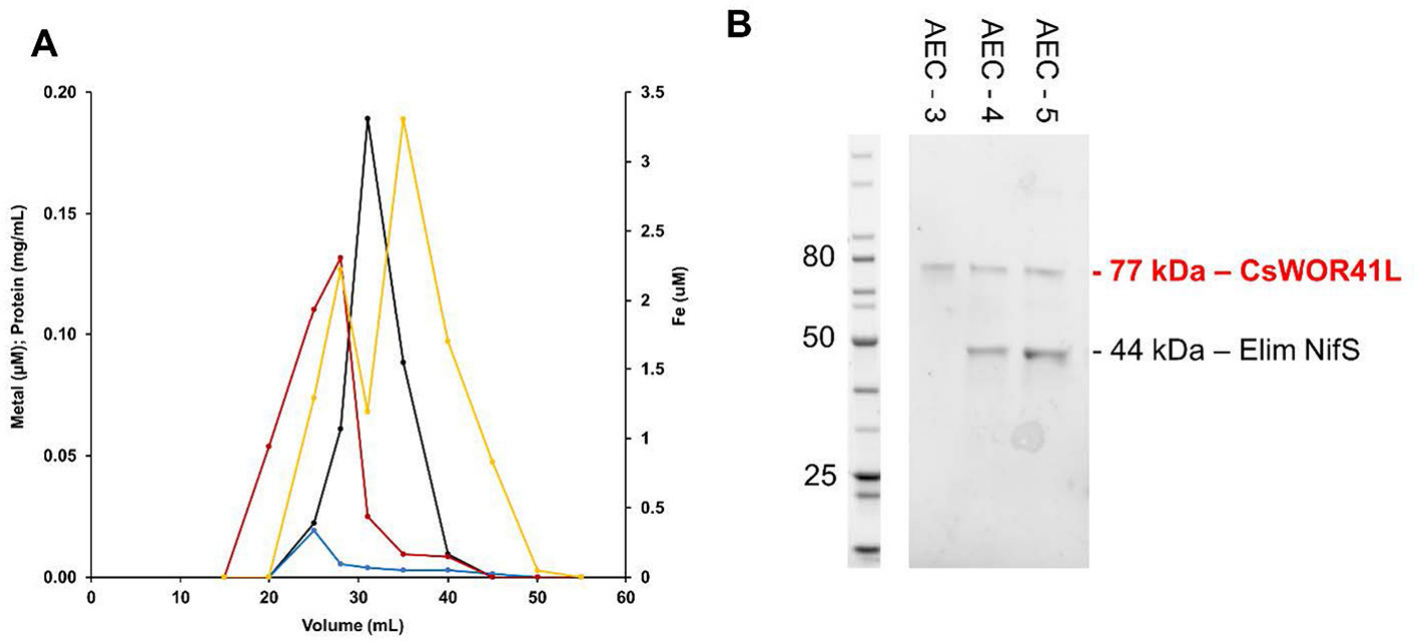
Purification of recombinant CsWOR41 using anion exchange chromotography. **(A)** Fractions from (Ni-NTA) chromatography column of heterologously expressed His-tagged CsWOR41LS in *E. limosum* containing W were combined and were separated on an anion exchange chromatography (QHP). Tungsten (red), molybdenum (blue), iron (yellow), and protein (black) concentrations in the QHP column fractions are shown. **(B)** Purified CsWOR41L (AEC3-5) was verified on an SDS-PAGE gel after QHP column.

To characterize CsWOR41, we recombinantly expressed the genes encoding the L and S subunits in *E. limosum*. Unlike *E. coli*, which contains a Class V WOR but not TupABC, *E. limosum* contains two WORs (ElWOR87 and ElWOR81), the Tup transporter and a Tuco biosynthesis pathway (Putumbaka et al., 2025b). *E. limosum* has an established genetic system (Shin et al., 2019; Sanford et al., 2023) and is therefore an ideal model organism to express and purify WOR family members. Since the function of CsWOR41X is unknown and it is not clear if it was a subunit of CsWOR41, we heterologously expressed the genes encoding the L and S subunits of CsWOR41 in *E. limosum* with a His-tag on the L subunit. Recombinant *E. limosum* was grown in the presence of 100 nM ${\text{MoO}}_{4}^{2-}$ and 100 nM ${\text{WO}}_{4}^{2-}$. CsWOR41LS was purified using a Ni-NTA affinity column and eluting fractions were analyzed by ICP-MS. The fractions containing W were pooled and further purified by QHP (Figure 6A). The single peak of W that eluted contained 5.0 nmol of W, 0.87 nmol of Mo and 60 nmol of Fe per mg of protein (Figure 6A). Only CsWOR41L, and not CsWOR41S, was identified in the W peak using LC-MS/MS. Analysis by SDS-PAGE (Figure 6B) revealed two major protein bands, one of which was determined by LC-MS/MS to be CsWOR41L at the expected size (77 kDa). The second major protein band was identified by LC-MS/MS as *E. limosum* cysteine desulfurase (IscS, 44 kDa), an unrelated enzyme that happens to co-purify. Partially purified CsWOR41L was tested for aldehyde oxidation activity using a plate assay to screen a total of 46 different aliphatic and aromatic aldehydes but no activity was detected. Nevertheless, ICP-MS data with purified WOR41L does show that it preferentially incorporates W and the partially purified enzyme contains approximately six times more W than Mo. Hence, these preliminary results with the Class V WORs from *C. somerae* and *E. coli* (Reschke et al., 2019) suggest that the diverse Clade 41 has both W- and Mo-containing enzymes depending on the organism.

### Expanding our understanding of WORs in the gut

Recent updates to the Unified Human Gastrointestinal Genome (UHGG) and Unified Human Gastrointestinal Proteome (UHGP) databases allows us to re-evalutate the abundance of WORs found in the human gut microbiome. Using the UHGP; v2.0.2 database, we found that 34 out of 92 clades had representatives found in the human gut microbiome (Supplementary Table S2). Unsurprisingly, the most abundant WORs in the gut microbiome are representatives of Clade 41 (~72%), which is biased due to the large number of *E. coli* sequences present (Supplementary Table S2). When the *E. coli* nodes are truncated and the abundance of WORs in the gut is normalized for the presence of Tup in the genome, 27 out of 92 clades are found in the human gut microbiome (Table 3). Clades 7, 81, 84, 85, and 87 are the most abundant in the gut microbiome when normalized for Tup, with Clades 7, 81, and 85 representing ~80%. Clade 7 is made up of both Class I and Class II WORs. Clade 81 has only Class II WORs. ElWOR81 is a member of clade 81, but no physiological function has been characterized for a clade 81 WOR so far. Clade 85 consists of only Class I WORs that are mainly found in *Desulfovibrio* species. Our understanding of these gut WORs is mainly limited to Clade 87, which contains ~10% of the WORs in the gut microbiome. Although Clade 87 contains characterized Class I, Class III, and Class IV WORs, the Class III WOR is not from a gut microorganism.

**Table 3:** Distribution of WORs by clade in the UHGP.

Clade	Total (%)
1	0.08
2	0.56
3	0.44
6	0.05
**7**	**19.24**
13	0.56
20	0.03
21	0.15
27	0.03
37	0.03
38	0.05
39	0.56
41	0.90
42	0.08
43	0.10
51	0.82
64	0.05
71	1.02
73	0.10
74	0.05
78	0.56
80	0.08
**81**	**32.58**
83	0.18
84	6.92
**85**	**24.90**
87	9.89
	**Total: 100**

The 27 WOR clades with representatives in the UHGP are listed. The abundance of each clade was normalized to organisms that also have TupABC, the tungstate specific transporter. The distribution of the WOR enzymes amongst the clades is reported as % of Total.

It is curious that some gut microbes may be utilizing W rather than Mo. In terms of supplements for human health, Mo is commonly found in daily vitamins but W is not. We assume the concentration of W in the gut is limiting considering the very low amount of W measured in foods (Schut et al., 2021). However, W utilizing organisms have systems in place to acquire and store W including Tup, a transporter with high affinity and specificity for tungstate, and the W storage protein Tub (Shao et al., 2025). Utilizing W in any environment may give an organism a competitive advantage over organisms that are competing for Mo. In fact, in the gut Mo is utilized by both gut microbes and the host because human cells have four Mo-containing enzymes. The ability of W-containing catalytic sites to carry out reactions of much lower potential than those containing Mo may also be an advanatage (Szaleniec and Heider, 2025). Clearly, much more research is needed to characterize the WOR family and particularly the ubiquitous and diverse Class V respiratory enzymes.

## Conclusions

The non-phylogenetic classification system of WOR enzymes based on predicted subunit composition and electron carrier specificity introduced here is helpful in identifying areas for future investigation. To date, four Class I, single-subunit Fd-dependent cytoplasmic WOR enzymes have been characterized from three different clades (including CsWOR87, characterized herein). Class I WORs oxidize aldehydes; in the gut microbiome, this is thought to be for detoxification of toxic aldehydes in foods or formed as metabolic byproducts (Schut et al., 2021). These four enzymes display a remarkable diversity in substrate specificity, biochemical properties, physiological function, and are found in both archaea and bacteria. They range from those with a broad aldehyde substrate specificity (PfAOR, BmWOR, CsWOR87) to those with a defined role in glycolysis (PfGAPOR), and from those that are highly oxygen-sensitive (PfAOR, PfGAPOR, CsWOR87) to those that are oxygen insensitive (BmWOR). These enzymes are found in diverse organisms including hyperthermophilic archaea (PfAOR, PfGAPOR) and mesophilic bacteria found in the human microbiome (BmWOR, CsWOR87) (Mukund and Adams, 1991, 1995; Thorgersen et al., 2022). If this diversity can be seen for enzymes representing 3 clades, how much further diversity exists in the other 52 clades containing the simplest of WOR Class I enzymes?

The more complex WOR classes further demonstrates the diversity of the enzyme family while still conserving the overall structure and cofactor content of the pyranopterin-containing WorL (Szaleniec and Heider, 2025). Characterized Class II enzymes include one with the same physiological role as Class I PfGAPOR (CbGOR) (Scott et al., 2019) and another that is active with aliphatic sulfonates (PfASOR) (Mathew et al., 2022). That leaves 38 clades containing Class II WORs with no characterized member. Even though Class III enzymes use NAD(P)^+^ as an electron carrier (instead of Fd, like Classes I and II) and Class IV enzymes use both NAD(P)^+^ and Fd in bifurcating reactions, structures of WORs from these classes are similar in that the LS subunits are shown to form a “nanowire” type structure that enables multilple electron transfers (Winiarska et al., 2023; Feng et al., 2025). Class IV WORs also include GmBCR showing that these enzymes can even have functions that are unrelated to aldehyde redox chemistry (Huwiler et al., 2019). The membrane associated Class V WORs found only in Clade 41 do not have a known physiological function as yet. Some members of this Class (YdhV, *E. coli*) (Reschke et al., 2019) that are found in microbes lacking the high affinity tungstate transporter TupABC, likely use Mo rather than W in the pyranopterin active site. In support of that conclusion, our characterization of heterologously expressed CsWOR41, from an organism that does contain TupABC, indicates this Class V WOR preferentially incorporates W. Further investigation into the biochemistry and physiological functions of WORs is clearly warranted and targets can be selected using both the WOR phylogenetic tree (92 clades) and the WOR operon system (five classes).

## Materials and methods

### Bacterial culture and media

*Cetobacterium somerae* ATCC-BAA-474 was obtained from the DSMZ-German Collection of Microorganisms and Cell Cultures in Braunschweig, Germany. The base growth medium consisted of 5 g/L glucose, 5 g/L brain heart infusion, 0.5 g/L yeast extract, 10 mM 3-(N-morpholino)propanesulfonic acid (MOPS), 1 mM KPO_4_, 9.3 mM NH_4_Cl, 4.4 mM KCl, 1.6 mM MgCl_2_•6H_2_O, 1.0 mM CaCl_2_•2H_2_O, 100 nM (NH_4_)_2_MoO_4_•4H_2_O, 100 nM Na_2_WO_4_•2H_2_O, 3 mM cysteine, 6 mM sodium bicarbonate, vitamin mix and trace elements as previously described (Bernalier et al., 1993; Schut et al., 2021). The pH of the medium was adjusted to pH 7.0 before filter sterilization using a 0.2 μM filter. *C. somerae* was routinely grown anaerobically with a headspace of 20% CO_2_ and 80% N_2_ at 35 °C with shaking (120 rpm). When grown in the 20 L fermenter, the same conditions were used. The fermenter was stirred at 150 rpm and was sparged with 20% CO_2_ and 80% N_2_ at a rate of 1.5 L/min.

### Gene context and class determination

A list of the more than 4,000 UniProt IDs used in the originally published phylogenetic tree was created (Schut et al., 2021). This list was uploaded into the EFI Gene Neighborhood Tool (EFI-GNT) (Zallot et al., 2019; Oberg et al., 2023) using the default parameter. The tool generated nearly 4,000 “gene neighborhoods” from each microorganism where the WorL gene is in the center flaked by 10 genes proceeding and 10 genes succeeding it (regardless of orientation). The results were downloaded as an SQLite DB from the EFI-GNT website. Using the DB Browser for SQLite (https://sqlitebrowser.org/), we created a database view joining “attributes” and “neighbors” data tables together using their indexes. The joined view was exported as CSV file and the data was transposed. The transposed file was loaded into Excel and conditional formatting was added to aid in the visual identification of potential operons and specific InterPro domains (Supplementary Dataset 1). Class I was defined by the presence of a single large subunit (WorL) containing a [4Fe-4S] cluster without adjacent subunits and having both domains (IPR013983 and IPR001203). Class II required a small subunit (WorS) containing four [4Fe-4S] clusters (IPR017896), while Class III was identified by an additional WorN subunit containing an FAD-binding domain (IPR023753) for NAD(P)^+^ interaction. Class IV WORs were identified as containing the WorABC subunits, which were identified through having the following IPR configurations: WorA (IPR001041 + IPR017896), WorB (IPR011538 + IPR019554 + IPR019575 + IPR017896), and WorC (IPR042128 + IPR041921). WorS-like genes that were also annotated as SAM-like proteins or WorN genes that also had ThiS designations were excluded, as these genes are most likely involved in pterin biosynthesis and are not WOR subunits. Class IV was determined by the presence of a five-subunit complex (WorLSABC) predicted to facilitate electron bifurcation. Class V enzymes were specifically characterized by a WorS subunit encoding a twin-arginine translocation (Tat) signal (IPR006311) and/or transmembrane domains, indicating cell membrane association. The original clade numbers were added to the file to allow for cross-referencing. The classifications were assigned manually based on this criteria.

### Phylogenetic tree

Based on the phylogenetic tree built using WorL (IPR013983 and IPR001203), 380 bacterial taxa belonging to 37 different genera encode members of clade 41 (Schut et al., 2021). Genomes, one for each genus, were selected based on quality and completion of genome sequence using the Bacterial and Viral Bioinformatics Resource Center (BV-BRC) preferentially selecting representative genomes where possible (Supplementary Dataset 3) (Olson et al., 2023). The genome sequence and annotation file for each selected genome was obtained from the National Center for Biotechnology Information (NCBI) (Sayers et al., 2022) and uploaded to a KBASE narrative (Arkin et al., 2018). A rooted phylogenetic species tree was constructed from the annotated genomes using the Insert Set of Genomes Into SpeciesTree – v2.2.0 app, which uses FastTree 2.0.0 and a set of 49 core genes to infer maximum-likelihood phylogenies (Price et al., 2010). The tree was visualized using Interactive Tree of Life v6.8.1 (Letunic and Bork, 2021). The presence or absence of *modA* and *tupA* for each genus was determined using protein Blast of known ModA and TupA protein sequences against taxonomic identifiers corresponding to the indicated genera (Supplementary Dataset 3) (States and Gish, 1994).

### Metal determination

Samples were diluted into 2% (v/v) trace-grade nitric acid (VWR) in acid-washed 15 mL polypropylene tubes. Samples were incubated at 37 °C temperature with shaking for at least 1 h and then centrifuged at 5,000 × *g* for 15 min to pellet debris. ICP-MS was done using an Agilent 7,900 ICP-MS fitted with MicroMist nebulizer, platinum cones, x-lens, and an Octopole Reaction System collision cell with He mode (Agilent Technologies). Results are reported as the average of three analytical replicates.

### Aldehyde oxidation plate assays

The 96-well plate aldehyde oxidation assays were performed under anaerobic conditions (5% H_2_, 95% Ar) in a Coy anaerobic chamber at 25 °C as previously described (Schut et al., 2021). Reaction mixtures (200 μL) contained 50 mM HEPES pH 7.5, 250 μM of the aldehyde or related substrate, 1 mM benzyl viologen, ~4 μM sodium dithionite and 1 to 100 μg cell extract or partially purified protein. For the 96-well plate aldehyde oxidation assay, aldehyde stock solutions (50 mM) were made in ethanol. A total of 46 aldehydes were used, as described in Schut et al. (2021). Assays were conducted at room temperature in a Coy anaerobic chamber (5% H_2_ and 95% Ar). A total of 20 μL each aldehyde stock solution was arrayed in a 2 mL deep 96-well plate to which 980 μL assay buffer (50 mM Hepes, pH 7.5) was added. A multichannel pipette was used to transfer 50 μL working substrate stock solutions to a clear flat-bottom Costar 96-well assay plate. A master mix was then prepared containing 10 mL anaerobic assay buffer, 1 mM benzyl viologen, enzyme (1 to 100 μg protein), and ~4 μM sodium dithionite (which resulted in a light blue color from a small amount of reduced benzyl viologen). At time 0, 150 μL master mix was multichannel pipetted into the assay plate, and the plate was covered with an airtight clear membrane before transferring out of the anaerobic chamber. The increase in absorbance at 600 nm compared to control assays in the absence of substrate was measured in a Molecular Devices Spectra Max 190 UV-Vis spectrophotometer. Assays for cytoplasmic extracts are reported as specific activities (μmoles of BV reduced/min/mg protein), while for partially purified enzymes they are reported as percent activity compared to that measured with the most active substrate. All assays were performed in triplicate and SDs between the replicates are reported.

### Heterologous expression of CsWOR41LS

The genes encoding CsWOR41L and CsWOR41S were cloned into pJIR plasmid (Shin et al., 2019) under control of an anhydrotetracycline inducible promoter. A His_6_-tag was added to the N-terminal of the L subunit. The plasmid with heterologous expressed CsWOR41LS was transformed into *E. limosum* wildtype strain and strains with chloramphenicol resistance were selected for and the plasmid was sequence verified. The *E. limosum* strain was then grown on 15 μg/mL thiamphenicol to select for the plasmid and 30 ng/mL anhydrotetracycline to induce expression (Shin et al., 2019).

## Data Availability

The datasets presented in this study can be found in online repositories. The names of the repository/repositories and accession number(s) can be found in the article/Supplementary material.
