# Supplementary material for: Classification of tungsten-containing oxidoreductases provides insights into their biochemical and physiological diversity
Source: Front Microbiol. 2026 Jun 19;17:1849799. doi: 10.3389/fmicb.2026.1849799 (PMC13328260; doi:10.3389/fmicb.2026.1849799)
Supplement: Supplementary file 4 [file Data_Sheet_1.pdf]

Frontiers in Microbiology

SUPPLEMENTARY INFORMATION

Classification of Tungsten-Containing Oxidoreductases Provides Insights into Their Physiological and Biochemical Diversity

Saisuki Putumbaka, Michael P. Thorgersen, Gerrit J. Schut, Farris L. Poole II, Claire E. Barrow, Jennifer B. Glass and Michael W. W. Adams

Department of Biochemistry & Molecular Biology, University of Georgia, Athens, GA 30602, USA

Supplementary Datasets 1-3
Supplementary Figures S1-S4
Supplementary Table S1-S2

Supplementary Dataset 1. Every WOR family member from the phylogenetic tree (Figure 1) and data from EFI-GNT with conditional formatting for each subunit. Provided as a separate Excel file.

Supplementary Dataset 2. WOR family members from the phylogenetic tree (Figure 1) that have been rendered obsolete sequences by InterPro and UniProt updates. Provided as a separate Excel file.

Supplementary Dataset 3. Data used for Clade 41 (Class V) phylogenetic tree analysis (**Fig. 5**). Provided as a separate Excel file.

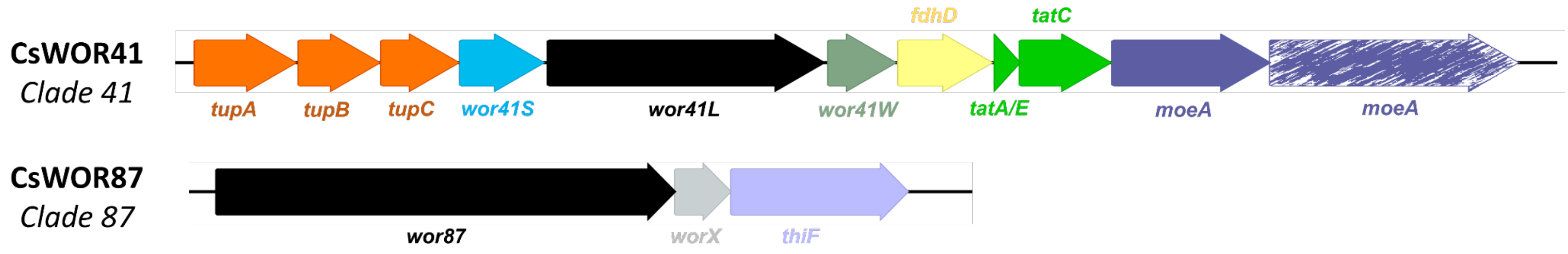

Figure S1. Genome context and operon structure of WORs in *C. somerae.* CsWOR41 (Clade 41) has two subunits: a large tungstopterin containing subunit, WOR41L (black) and a small subunit, WOR41S (blue), containing four [4Fe-4S] clusters. Also shown is a potential subunit of unknown function, WOR41W (green). Upstream there are genes encoding the tungstate-specific transporter, Tup (orange), and downstream there is an *fdhD*-like gene (purple), two Tat signal related transport and cleavage genes (bright green) and two genes encoding MoeA (purple), which is involved in pterin biosynthesis. CsWOR87 is a single subunit protein (WOR87L, black). Downstream there are genes encoding WorX (grey) and ThiF (purple) which are thought to be involved in the pterin biosynthesis pathway and involved in processing and binding of pterin to the enzyme.

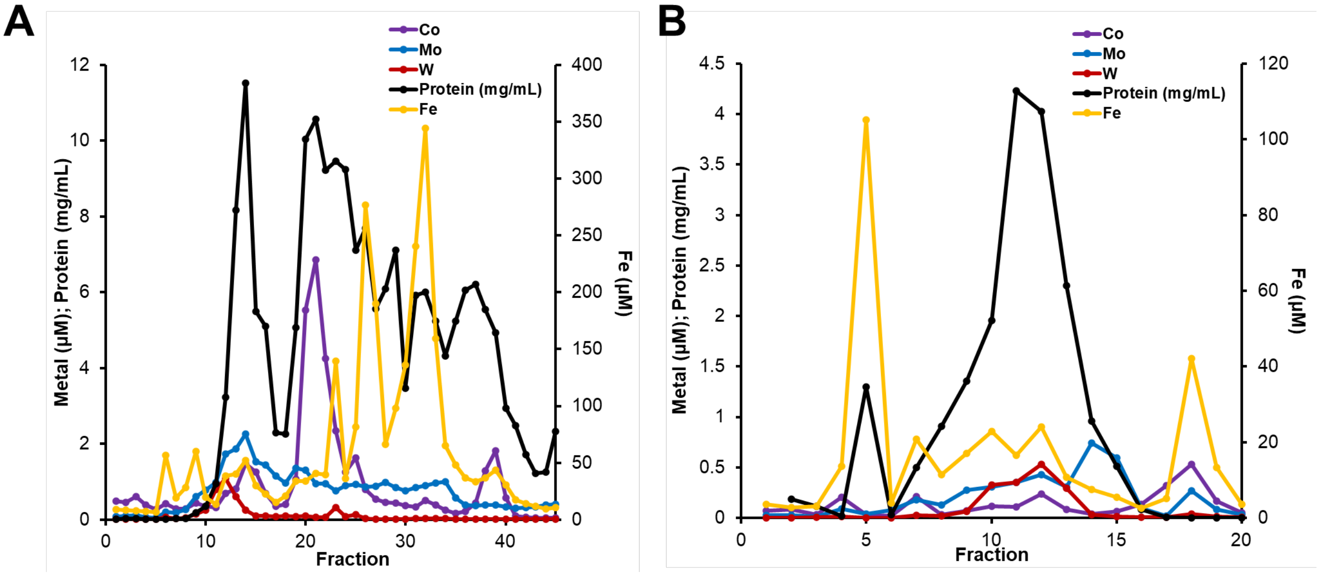

Figure S2. **Metal content of *C. somerae* cytoplasmic extract after column chromatography.** ICP-MS metal analyses for cobalt (Co; purple), molybdenum (Mo; blue), tungsten (W; red), iron (Fe; yellow) are shown, along with the protein concentration (mg/mL; black) present in fractions from A) cytoplasmic extract separated using a QHP column and B) subsequent separation of QHP fractions 12-14 on a size exclusion column (S200). Concentrations of Fe are shown on right axis while those of the other metals (Co, Mo and W) and of protein are shown on the left axis.

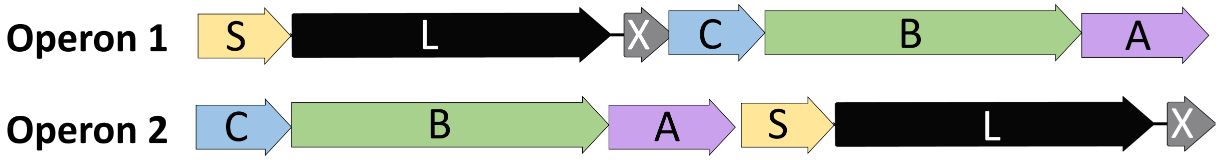

Figure S3. Two operon structures for Class IV enzymes. Operon 1 has *worSL* followed by *worCBA* and operon 2 has *worCBA* followed by *worSL*. The *worX* gene is not present in all operons encoding Class IV WORs.

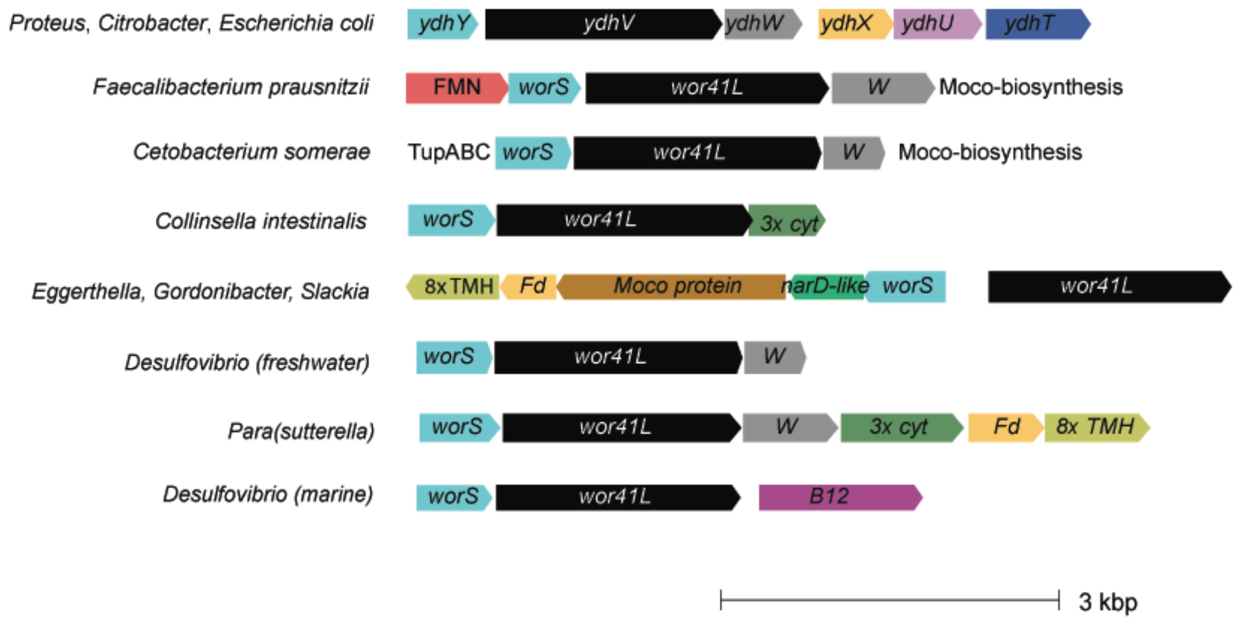

Figure S4. Gene context of various types of Class V WORs. Class V WORs are found only in clade 41 and the putative operons all encode the large tungstopyranopterin-containing WorL subunit (black) the small WorS subunit that contains four 4Fe-4S clusters with a Tat signal (blue). Other subunit compositions and gene context varies greatly as displayed for various groups of Bacteria.

Table S1. Relative activity of partially purified CsWOR87 from fraction 13 (**Fig 3B**) with aliphatic and aromatic aldehydes. Activity was screened with aldehydes (column 1) that have been found in the gut microbiome (blue), aldehydes that have been found in cooked foods (orange) and aldehydes for which the corresponding acid has been found in the gut microbiome (green). Relative activity is reported as a relative percentage to the substrate that had the highest activity, p-Tolualdehyde, as measured by the absorbance at OD_{600} of benzyl viologen.

**Substrate**	***C. somerae* WOR87 (%)**
p-Tolualdehyde	100
Salicylaldehyde	100
Glyceraldehyde Dimer	98
Butyraldehyde	79
Isobutyraldehyde	78
Benzaldehyde	76
2-Furaldehyde	69
Phenylacetaldehyde	68
4-Hydroxy-3-methoxycinnamaldehyde	63
Pentanal	63
Propionaldehyde	53
Vanillin	50
Acetaldehyde	49
2-Phenylpropionaldehyde	45
3-Methyl-2-butenal	38
2-Methyl-2-butenal	29
Terephthaldehyde	25
2-Methylbutyraldehyde	24
4-Hydroxybenzaldehyde	21
Phenylpropionaldehyde	20
Isovaleraldehyde	16
2-Ethylisovaleraldehyde	15

Table S2. Distribution of WORs by clade in the UHGP. The 27 WOR clades with representatives in the UHGP. In contrast to Table 3, the abundance of each clade is not normalized to organisms that also have TupABC, the tungstate specific transporter. The distribution of the WOR enzymes amongst the clades is reported as % of the total (> 10% indicated in **bold**).

Clade	Total (%)
1	0.47
2	0.95
3	0.24
6	0.24
7	5.67
13	0.24
14	0.47
20	0.24
21	0.47
27	0.24
29	0.47
30	0.24
37	4.49
38	0.47
39	0.24
40	0.24
41	**41.37**
42	2.60
43	0.71
45	0.71
46	3.55
51	0.24
53	0.24
64	0.47
71	1.65
73	0.71
74	0.24
78	3.07
80	0.47
81	**17.26**
83	1.42
84	2.60
85	8.04
87	**18.91**
